# “Physiological and demographic responses of *Nilaparvata lugens* to combined climate stressors: CO_2_, temperature, and ozone”

**DOI:** 10.3389/fpls.2025.1518361

**Published:** 2025-06-05

**Authors:** Yogesh Yele, Subhash Chander, Sachin S. Suroshe, Prabhulinga Tenguri, Arya Pattathanam Sundaran, G. Guru Pirasanna Pandi, Arti Bhatia, Chenesh Patel

**Affiliations:** ^1^ ICAR-National Institute of Biotic Stress Management, Raipur, India; ^2^ ICAR-National Center for Integrated Pest Management, New Delhi, India; ^3^ ICAR-Indian Agricultural Research Institute, New Delhi, India; ^4^ ICAR-Natioal Bureau of Agricultural Insects Resources, Bengaluru, India; ^5^ ICAR-National Rice Research Institute, Cuttack, India; ^6^ Department of Entomology, DKS, College of Agriculture and Technology, Bhatapara, Chhattisgarh, India

**Keywords:** brown planthopper, climate change, elevated ozone, insects, pest management, rice

## Abstract

Climate change factors, including elevated carbon dioxide (eCO_2_), elevated ozone (e0_3_), and the combined effect of elevated temperature and CO_2_ (eT+eCO_2_), significantly influence the population dynamics, development, and feeding behavior of the Brown Planthopper (*Nilaparvata lugens*, BPH) and its impact on rice yield. A two-year field study (2019–2020) showed that BPH populations were highest under eCO_2_ (61.6 ± 13.5 and 50.6 ± 12.3 N*. lugens*/hill) and moderate under eT+eCO_2_ (44.5 ± 9.4 and 47.5 ± 12.1 N*. lugens*/hill), while e0_3_ drastically reduced populations (17.7 ± 3.1 and 25.1 ± 7.0 N*. lugens*/hill). Fecundity followed a similar trend, with the highest egg production under eCO_2_ (219.7 ± 3.3 and 234.3 ± 9.7 eggs/female), moderate under eT+eCO_2_ (194.2 ± 6.3 and 223.5 ± 9.2 eggs/female), and lowest under e0_3_ (108.4 ± 6.0 and 135.6 ± 3.7 eggs/female). Developmental duration was shortest under eT+eCO_2_ (14.9 ± 0.3 and 15.9 ± 0.4 days) and longest under e0_3_ (18.2 ± 0.40 and 21.7 ± 0.40 days). Feeding intensity, indicated by honeydew excretion, was highest under eCO_2_ (124.8 ± 5.3 and 131.3 ± 4.2 mm²), reduced under eT+eCO_2_ (105.7 ± 4.9 and 107.6 ± 3.4 mm²), and lowest under e0_3_ (44.2 ± 2.5 and 48.9 ± 2.6 mm²). Results indicated that eCO_2_ promoted overall plant growth, with the highest plant height (65.4 ± 0.8 cm) and reproductive tillers (22.2 ± 0.6). However, under BPH infestation, eCO_2_ also resulted in the highest yield reduction (15.9%) despite producing the highest grain yield under uninfested conditions (40.1 ± 0.3 g/hill). The eT+eCO_2_ treatment exhibited moderate reductions in plant height (62.4 ± 0.6 cm) and grain yield (38.1 ± 0.4 g/hill), with a yield loss of 11.5% under infestation. The e0_3_ treatment negatively impacted plant growth, significantly reducing plant height (54.8 ± 1.0 cm), total tillers (17.7 ± 0.9), and grain yield (27.5 ± 0.2 g/hill) in uninfested conditions, with a lower yield reduction (8.72%) under infestation. The findings of this study indicate that pests and host plants benefited under eCO_2_ and eT+eCO_2_ conditions; however, increasing BPH populations caused yield losses. Nevertheless, e0_3_ had a detrimental effect on pests as well as host plants. The results pertaining to the collective impact of climate change factors on both the host plant and pests have the potential to contribute to the advancement of insect pest management strategies in response to shifting climates.

## 1 Introduction

Over half of the world’s population receives nutrition from rice (*Oryza sativa* L.), making it the most significant staple food in the world (Khush, 2004). In the fiscal year 2023, India is expected to have produced more than 135 million metric tons of rice. This indicated that rice production has been rising steadily since 2017. In India, rice is a staple food that is consumed on a large scale. The area planted to scented rice varieties, particularly Basmati, is increasing year on year due to both domestic and international demand. However, because of a number of biotic and abiotic factors, rice productivity is declining in India (Behura et al., 2011). Despite this, the country’s population boom will cause a continued increase in the demand for grain in the ensuing decades. The brown plant hopper (BPH), *Nilaparvata lugens* (Stal.) outbreaks have happened throughout rice cultivation history, but with the advent of green revolution, improved rice varieties and input intensive farm practices, the outbreaks became more frequent and intense. *N. lugens* causes direct damage to the rice plant by sucking the phloem sap, causing them to wilt and completely dry out- a phenomenon known as “hopper burn”, inflicting a yield loss of 70% (Krishnaiah et al., 2008) and transmits viral diseases such as grassy stunt and ragged stunt virus.

The incidence of herbivorous insect pests and crop yield are both affected by major climate change factors, such as increased atmospheric CO_2_ concentration, rising temperatures, and elevated tropospheric ozone levels (Raderschall et al., 2021; IPCC, 2022). By 2050, atmospheric CO_2_ levels are anticipated to attain 550 ppm as a result of the increase in anthropogenic greenhouse gas emissions. This acceleration is anticipated to result in a global temperature increase of 1.8 to 4°C by the century’s end (IPCC, 2022). Similarly the concentration of ground level ozone have enhanced significantly since the industrial revolution (Morgan et al., 2006), and have risen from 10 ppb in late 1800s to average levels of 40 ppb currently (Brauer et al., 2016).

The effects of elevated CO_2_ and temperature on plant physiology and phytochemistry are well-documented (Ainsworth et al., 2007). Elevated CO_2_ increases leaf mass per area and the C:N ratio by promoting carbohydrate accumulation while diluting nitrogen content (Coley et al., 2002). In contrast, elevated temperatures enhance leaf biomass and nitrogen content (Way and Oren, 2010). In context of insect, elevated CO_2_ indirectly affects insects by driving herbivores to feed more nutrient-deficient plants to meet nitrogen demands resulting in population expansion (Srinivasa Rao et al., 2009). A similar outcome was observed in the wheat aphid *Sitobion avenae*, which showed a significant increase under elevated CO_2_ conditions compared to ambient levels (Chen et al., 2004). Elevated temperatures are likely to directly affect insect development by raising metabolic rates, which shortens development (Bale et al., 2002). Numerous investigations have indicated that the duration of various aphid instars decreased with rising temperatures (Wang and Tsai, 2001). Elevated ozone level affects insect fitness by modifying growth rate, developmental duration, survival, feeding behavior, and oviposition (Ward and Masters, 2007; Brownlie and Johnson, 2009). The impacts may be positive, negative, or neutral (Couture and Lindroth, 2012; Capone et al., 2013). It is typically not directly associated with the nutritional attributes of plants. Elevated CO_2_, temperature, and ozone levels may have a direct impact on agricultural production as well as an indirect impact due to insect pests (Coakley et al., 1999). Previous research suggests that rice agriculture faces considerable threats from anticipated environmental changes (Long, 2012).

Most research has examined the individual effects of elevated CO_2_, temperature, and ozone on crop yields and crop growth in controlled environments. However, studies on their interactive effects CO_2_ & temperature, and ozone remain scarce. In the Indian context, research on climate change’s impact on crop-pest interactions is even more limited. Given this gap, it is crucial to assess how rising CO_2_, temperature, and ozone influence rice crops and their major sucking pest, the brown planthopper (BPH).

## 2 Materials and methods

### 2.1 Collection and maintenance of *N. lugens*

The BPH population (nymphs and adults) was collected from unsprayed rice fields of ICAR-Indian Agricultural Research Institute (IARI), Research Farm (28°64 N, 77°17 E and 228.61 m). Thereafter, the BPH population was transferred to a glass house at the Division of Entomology, IARI, New Delhi, under controlled conditions of temperature (28 ± 2°C), relative humidity (70 ± 5%), and photoperiod (14L:10D) on a susceptible rice variety, Taichung Native 1, in order to facilitate mass multiplication. To obtain a homogeneous BPH population, ten pairs of adults (males and females) were placed onto uninfested rice pots (40 days old), allowed 24 hours to oviposit, and then employed for further study (Babu et al., 2022).

### 2.2 Experimental setup

The experiments were carried out at the Free Air Temperature Enrichment (FATE) facility of the Division of Environmental Science, ICAR-IARI, New Delhi, during the rainy season from July to October in 2019 and 2020. Each FATE ring had a circular area of 28 m^2^ and the elevated CO_2_ level was maintained only during the day time. CO_2_ concentration was measured by Infra-Red Gas Analyzer (IRGA, PP system, SBA-5) placed at the center of each ring. The temperature inside the FATE rings was elevated using infra-red heaters. O_3_ was produced by an ozone generator (Eltech Engineers, Mumbai) by converting atmospheric oxygen into O_3_ using UV lamps and its concentration inside the rings was monitored by an ozone analyzer (2B Technologies). Four FATE rings were used to conduct the experiments. Ring one had an elevated carbon dioxide (eCO_2_) condition of 600 ± 25 ppm. Ring two was equipped with elevated temperature (Ambient+3°C) and carbon dioxide (600 ± 25 ppm) (eT+eCO_2_) conditions. Ring three was equipped with only elevated ozone (65 ± 5 ppb) (e0_3_) conditions. Ring number four was used as a control with ambient temperature, CO_2_ and O_3_ conditions (AM). Hereafter, all four exposure conditions will be referred to as eT+eCO_2_, eCO_2_, e0_3_, and AM throughout the manuscript. Rice plants (Pusa Basmati 1121) were grown under these conditions and then insects were released to assess the effect of eT+eCO_2_; eCO_2_; e0_3_ and AM on *N. lugens* development and survival along with yield parameters.

### 2.3 Study of demographic structure of *N. lugens*

To study the population structure of *N. lugens*, 25-day-old potted rice plants were transferred to all four FATE rings (eT+eCO_2_, eCO_2_, e0_3_, and AM) and enclosed in Mylar cages with two windows for aeration. After ten days of crop exposure to each condition, five pairs of gravid brachypterous females and males were released into 10 replications in each treatment (Pandi et al., 2018a). The number of nymphs, males, and females per hill was recorded weekly in 2019 and 2020 for BPH demographic study.

### 2.4 Study on biological parameters of *N. lugens*

In this experiment we studied development duration, adult longevity and fecundity. First instars of *N. lugens* nymphs were released in each exposure treatment in order to analyze the nymphal period. The nymphal duration for each instar and the total nymphal period were calculated based on the moulting data of *N. lugens* (Pandi et al., 2018a). Adult lifespan was assessed by transferring newly emerged males and females to each treatment conditions and recording their longevity till their death. To assess fecundity, a freshly emerged pair, consisting of a brachypterous female and a winged male, was introduced into each exposure treatment for mating and egg-laying (Cheng et al., 2001). After seven days, the leaf sheath of rice plant was carefully removed using fine forceps and a scalpel. The exposed inner tissue was examined under a stereomicroscope (40× magnification) to visualize the embedded eggs. The total number of eggs per plant was counted manually using a fine needle to mark the counted eggs (Horgan et al., 2021). Experiment consisted of 10 replications and was carried out throughout two rainy seasons during 2019 and 2020.

### 2.5 Feeding potential of *N. lugens* by honeydew test

Feeding potential of *N. lugens* was assessed by measuring the honeydew excreted by newly emerged females on Whatman No. 1 filter paper treated with a 0.5% bromocresol solution. After oven-drying at 100°C for 5 minutes, the honeydew stains developed a violet or purple color due to their amino acid content. The excreted spots were traced onto tracing paper, and their areas were quantified using millimeter-scale graph paper by counting the enclosed squares (Paguia et al., 1980). Each treatment was replicated ten times across two consecutive seasons to ensure the reliability of the results.

### 2.6 Plant parameters

To study plant parameters viz., plant height, number of tillers, reproductive tillers, panicle length, grain per panicle, test weight, and yield per hill were recorded for each treatment.

### 2.7 Statistical analysis

The data of *N. lugens* population dynamics were normalized using the square root transformation and subjected to two-way analysis of variance (ANOVA). F-tests were used to determine the significance of differences between treatments and weeks tested by F- tests, while least significant differences (LSD) were used to compare treatment means at P=0.05. Data on biological parameters of *N. lugens* and plant parameters were subjected to analysis of variance (ANOVA), and the significance of differences between the treatments was tested by F-tests, while the treatment means were compared by the least significant differences (LSD) at P=0.05 using the statistical software SAS version 9.2.

## 3 Results

### 3.1 Population dynamics of *N. lugens* in FATE

**Figure 1:**
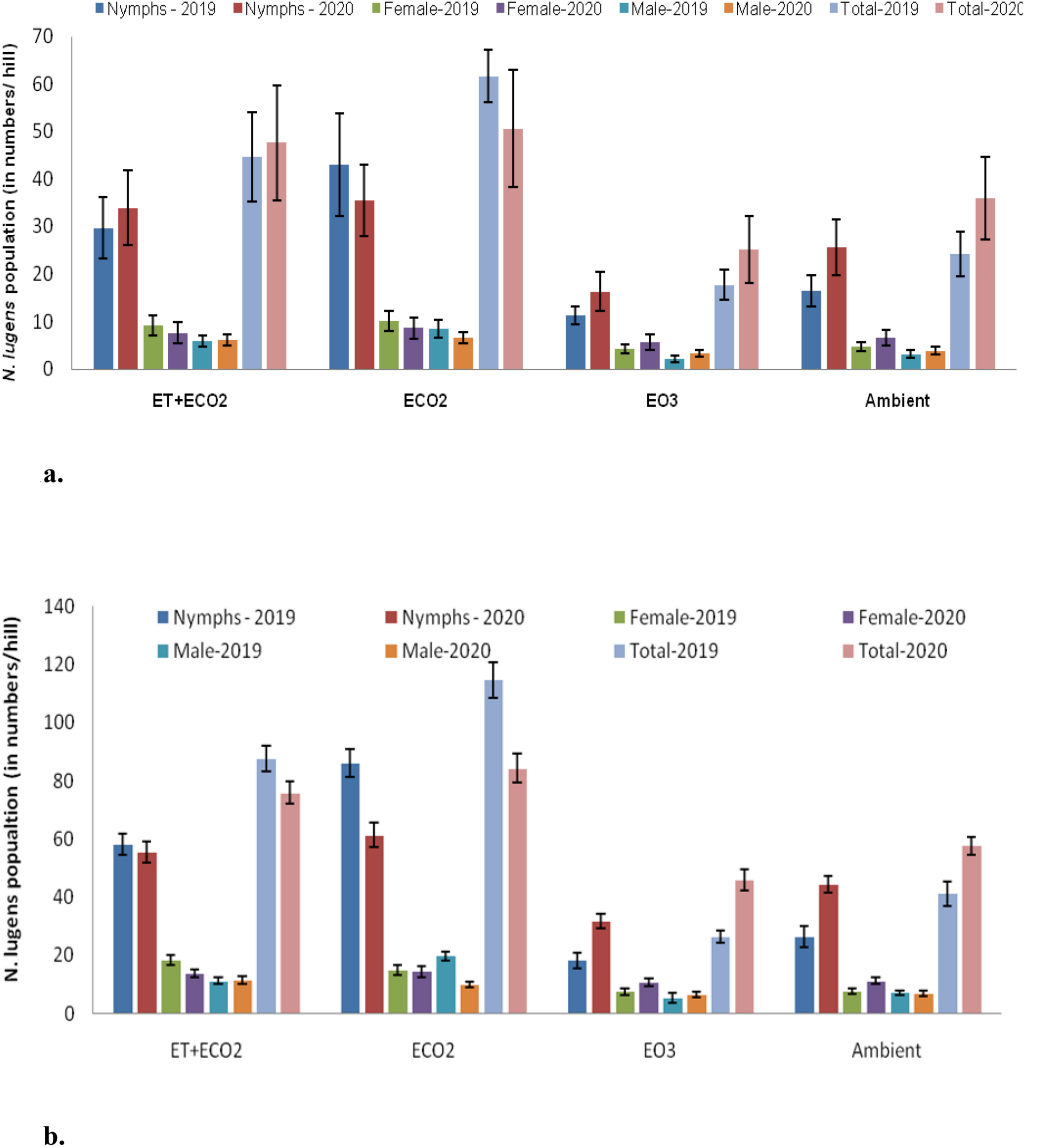
**(a)** Mean population of different stages of brown planthopper *N. lugens* recorded during the rainy season of 2019 and 2020. **(b)** Peak emergence of different stages of brown planthopper *N. lugens* recorded during the rainy season of 2019 and 2020.

*N. lugens* populations significantly differed across treatments (*F*=373.5, *P*<0.001 for 2019; *F*=113.19, *P*<0.001 for 2020), weeks (*F*=313.6, *P*<0.001 for 2019; F=450.7, P<0.001 for 2020), and treatment-week interactions (*F*=14.8, *P*<0.001 for 2019; *F*=5.6, *P*<0.001 for 2020), as shown in **Supplementary Tables 1**, **5**. In both 2019 and 2020, the population of *N. lugens* was higher in eCO_2_, followed by eT+eCO_2_, and lowest in e0_3_ when compared to the ambient population. In 2019, two peaks were recorded in eCO_2_: first in the 5^th^ weeks after release (WAR) (114.6 ± 6.2 N*. lugens*/hill) and second in the 8^th^ WAR (100.7 ± 5.6 N*. lugens*/hill) whereas in 2020, first in the 3^rd^ WAR (72.2 ± 3.4 N*. lugens*/hill) and second in the 4^th^ WAR (84.3 ± 5.0 N*. lugens*/hill). In 2019 and 2020, eCO_2_ had the highest mean population density (61.6 ± 13.5 and 50.6 ± 12.3 N*. lugens*/hill) compared to the other treatments (**Figures 1a, b**). Conversely, the e0_3_ had the lowest average population density of *N. lugens* per hill, with observed values of 17.7 ± 3.1 and 25.1 ± 7.0 for the years 2019 and 2020, respectively. During 2019 and 2020, under eT+eCO_2_, average population density of *N. lugens* (44.5 ± 9.4 and 47.5 ± 12.1 N*. lugens*/hill) found to be significantly higher than ambient control but was significantly lower than eCO_2_ (**Figures 1a, b**). Furthermore, there were significant variations in the populations of nymphs and adults (male and female) among the treatments during both seasons (**Figures 1a, b** **;** **Supplementary Tables 2**, **3**, **6**, **7**). In both seasons, *N. lugens* nymphs and adults are most abundant in the eCO_2_, followed by the eT+eCO_2_, AM, and e0_3_ (**Figures 1a, b**). On the other hand, e0_3_ reduced the population of *N. lugens* and impeded rice crop development.

### 3.2 Biological parameters of *N. lugens* in FATE

#### 3.2.1 Fecundity and related parameters of *N. lugens*

The fecundity of *N. lugens* on rice plants showed significant difference among treatments in both years (F=90.1, P<0.001 and F=42.5, P<0.001). Females deposited the most eggs on rice plants exposed to eCO_2_ (219.7 ± 3.3 & 234.3 ± 9.7) followed by eT+eCO_2_ (194.2 ± 6.3 & 223.5 ± 9.2) and AM (128.6 & 163.1) in both years 2019 and 2020 (**Table 1**). In contrast to other treatments and the control, *N. lugens* fertility dramatically dropped on e0_3_-treated plants (108.4 ± 6.0 and 135.6 ± 3.7 eggs/female) in both seasons. In 2019 and 2020, rice plants that were exposed under eCO_2_ exhibited the highest number of eggs per female (16.9 ± 0.2 & 14.5 ± 0.4), while plants that were exposed under e0_3_ exhibited the lowest number of eggs per female (10.4 ± 0.3 & 10.0 ± 0.3). Egg masses under eT+eCO_2_ (13.5 ± 0.4 &12.6 ± 0.4) were considerably greater than ambient (11.1 ± 0.3 & 11.6), but lower than eCO_2_ in both the seasons.

**Table 1:** The combined effect of elevated temperature+CO_2_ and ozone on *N. lugens* fecundity and egg masses during the rainy season of 2019 and 2020.

Treatment	2019		2020	
	No. of egg masses	Fecundity	No. of egg masses	Fecundity
eCO_2_	16.9 ± 0.2^a^	219.7 ± 3.3^a^	14.5 ± 0.4^a^	234.3 ± 9.7^a^
eT+eCO_2_	13.5 ± 0.4^b^	194.2 ± 6.3^b^	12.6 ± 0.4^b^	223.5 ± 9.2^a^
e0_3_	10.4 ± 0.3^d^	108.4 ± 6.0^d^	10.0 ± 0.3^d^	135.6 ± 3.7^c^
AM	11.1 ± 0.3^c^	128.6 ± 5.9^c^	11.6 ± 0.4^c^	163.1 ± 4.1^b^
***F-value***	80.0	90.1	23.1	42.5
***P-value***	<0.001	<0.001	<0.001	<0.001

Data were mean of ten replications; Numbers with the same superscript in a column do not differ significantly (*P*<0.001).

F-value, test statistic from ANOVA indicating variance among treatments; p-value, probability value indicating statistical significance of differences among treatments (values <0.001 denote highly significant differences).

#### 3.2.2 Developmental parameters of *N. lugens*

Total nymphal duration, adult longevity and developmental period differed significantly across the treatments and seasons (**Tables 2A–C**). In both 2019 and 2020, the total nymphal duration was considerably shorter on plants grown under eT+eCO_2_ (14.9 ± 0.3 & 15.9 ± 0.4 days) compared to other treatments such as eCO_2_ (15.5 ± 0.17 & 17.3 ± 0.42 days), e0_3_ (18.2 ± 0.40 & 21.7 ± 0.40 days), and ambient (16.7 ± 0.15 & 18.8 ± 0.84 days). The total developmental period was significantly shorter under eT+eCO_2_ and eCO_2_ than ambient conditions, but no difference was observed between e0_3_ and ambient conditions in both season (**Tables 2A, B**). In comparison to ambient condition, stress condition drastically shortens the longevity of both sexes (**Table 2C**). In 2019, female and male longevity was lowest in eT+eCO_2_ (9.7 ± 0.3&8.7 ± 0.1days). In contrast, during 2020, e0_3_ had the lowest female and male longevity (11.1 ± 0.2 & 9.5 ± 0.5 days).

**Table 2a:** The combined effect of elevated temperature+CO_2_ and ozone on developmental stages of *N. lugens* in Free Air Temperature Enrichment (FATE) during the rainy season 2019.

Treatments	Nymphal Duration (days)					Total Nymphal (Days)	Adult longevity (Days)	Total developmental period (Days)
	I	II	III	IV	V			
eCO_2_	3.6 ± 0.16^b^	3.0 ± 0.00^b^	2.9 ± 0.10^a^	2.9 ± 0.10^a^	3.1 ± 0.10^b^	15.5 ± 0.17^c^	9.7 ± 0.40^b^	25.2 ± 0.53^b^
eT+eCO_2_	3.1 ± 018^c^	3.1 ± 0.1^b^	2.9 ± 0.10^a^	2.8 ± 0.13^a^	3.0 ± 0.15^b^	14.9 ± 0.31^c^	9.3 ± 0.33^b^	24.2 ± 0.55^b^
e0_3_	4.5 ± 0.17^a^	3.5 ± 0.17^a^	3.1 ± 0.10^a^	3.1 ± 0.10^a^	4.0 ± 0.20^a^	18.2 ± 0.40^a^	9.6 ± 0.27^b^	27.8 ± 0.55^a^
AM	3.8 ± 0.13^b^	3.7 ± 0.15^a^	3.0 ± 0.00^a^	2.8 ± 0.13^a^	3.4 ± 0.16^b^	16.7 ± 0.15^b^	11.8 ± 0.30^a^	28.5 ± 0.50^a^
***F-value***	12.8	7.1	1.2	1.4	7.8	21.7	11.2	14.6
***P-value***	<0.001	0.316	0.247	<0.001	<0.001	<0.001	<0.001	<0.001

Data were mean of ten replications; Numbers with the same superscript in a column do not differ significantly (*P*<0.001).

F-value, test statistic from ANOVA indicating variance among treatments; p-value, probability value indicating statistical significance of differences among treatments (values <0.001 denote highly significant differences).

**Table 2b:** The combined effect of elevated temperature+CO_2_ and ozone on developmental stages of *N. lugens* in Free Air Temperature Enrichment (FATE) during the rainy season 2020.

Treatments	Nymphal Duration (Days)					Total Nymphal (Days)	Adult longevity (Days)	Total developmental period (Days)
	I	II	III	IV	V			
eCO_2_	4.0 ± 0.21^b^	3.6 ± 0.22^b^	3.2 ± 0.13^a^	3.0 ± 0.21^a^	3.5 ± 0.17^b^	17.3 ± 0.42^c^	11.1 ± 0.43^b^	28.4 ± 0.73^b^
eT+eCO_2_	3.6 ± 0.16^b^	3.4 ± 0.16^b^	2.9 ± 0.23^a^	2.7 ± 0.15^a^	3.3 ± 0.15^b^	15.9 ± 0.46^c^	10.8 ± 0.33^b^	26.7 ± 0.68^b^
e0_3_	5.3 ± 0.21^a^	4.8 ± 0.20^a^	3.7 ± 0.21^a^	3.4 ± 0.16^a^	4.5 ± 0.31^a^	21.7 ± 0.40^a^	10.6 ± 0.50^b^	32.3 ± 0.78^a^
AM	4.5 ± 0.22^b^	4.0 ± 0.26^b^	3.4 ± 0.16^a^	3.1 ± 0.18^a^	3.8 ± 0.25^b^	18.8 ± 0.84^b^	12.6 ± 0.37^a^	31.4 ± 1.10^a^
***F-value***	12.8	8.4	3.1	2.6	5.3	19.7	4.8	9.6
***P-value***	<0.001	<0.001	0.037	0.065	0.004	<0.001	0.006	<0.001

Data were mean of ten replications; Numbers with the same superscript in a column do not differ significantly (*P*<0.001).

F-value, test statistic from ANOVA indicating variance among treatments; p-value, probability value indicating statistical significance of differences among treatments (values <0.001 denote highly significant differences).

**Table 2c:** The combined effect of elevated temperature+CO_2_ and ozone on *N. lugens* longevity during the rainy season of 2019 and 2020.

Treatments	Female longevity (Days)		Male longevity (Days)	
	2019	2020	2019	2020
eCO_2_	11.2 ± 0.2^b^	12.5 ± 0.2^b^	8.7 ± 0.1^b^	10.2 ± 0.2^b^
eT+eCO_2_	9.7 ± 0.3^c^	11.5 ± 0.1^c^	8.7 ± 0.1^b^	10.0 ± 0.2^b^
e0_3_	10.1 ± 0.2^c^	11.1 ± 0.2^c^	9.3 ± 0.3^b^	9.5 ± 0.5^b^
AM	12.5 ± 0.1^a^	13.3 ± 0.2^a^	10.1 ± 0.2^a^	11.5 ± 0.2^a^
***F-value***	22.6	19.7	8.2	6.4
***P-value***	<0.001	<0.001	<0.001	<0.001

Data were mean of ten replications; Numbers with the same superscript in a column do not differ significantly (*P*<0.001).

F-value, test statistic from ANOVA indicating variance among treatments; p-value, probability value indicating statistical significance of differences among treatments (values <0.001 denote highly significant differences).

#### 3.2.3 Feeding potential of *N. lugens* by honeydew test

The amount of honeydew excreted by *N. lugens* females differed significantly across treatments in both seasons (*F*=79.7, *P*<0.001in 2019 and *F*=145.1, *P*<0.001in 2020) (**Table 3**). During both seasons, brachypterous females fed on eCO_2_-exposed rice plants had a higher percent increase in honey dew excretion above ambient (224 and 229%), but e0_3_-exposed rice plants had a decrease percentage of honey dew excretion (-14 to 20%). In both years of study, females fed on ET+ EC exposed rice plants excreted much more honeydew than AM and e0_3_, but significantly less than eCO_2_ (**Table 3**).

**Table 3:** The combined effect of elevated temperature + CO_2_ and ozone on honeydew excretion during the rainy season of 2019 and 2020.

Treatment	Honeydew excretion (mm^2^)			
	2019	(%) increase/decrease over ambient in 2019	2020	(%) increase/decrease over Ambient in 2020
eCO_2_	124.8 ± 5.3^a^	224.4	131.3 ± 4.2^a^	229.5
eT+eCO_2_	105.7 ± 4.9^b^	190.1	107.6 ± 3.4^b^	188.1
e0_3_	44.2 ± 2.5^c^	-20.5	48.9 ± 2.6^c^	-14.5
AM	55.6 ± 3.9^c^	–	57.2 ± 2.5^c^	–
***F-value***	79.7	–	145.1	–
***P-value***	<0.001		<0.001	

Data were mean of five replications. Numbers with the same superscripts do not differ.

F-value, test statistic from ANOVA indicating variance among treatments; p-value, probability value indicating statistical significance of differences among treatments (values <0.001 denote highly significant differences).

### 3.3 Impact of climate change parameters on plant growth and yield of rice in FATE

The present study demonstrated that the *N. lugens* infestation had a deleterious effect on plant parameters in all treatments, including ambient. As a result, all plant parameters are much better under BPH-uninfested than infested conditions. In both infested and uninfested conditions, eCO_2_ improved plant parameters whereas e0_3_ significantly reduced them in contrast to eT+eCO_2_ and ambient (**Table 4**). Therefore, under uninfested condition the plant parameters such as plant height (65.4 ± 0.8cm), total tillers (25.0 ± 0.8), reproductive tillers (22.2 ± 0.6) and percent reproductive tillers (88.9 ± 1.0%) were recorded maximum in eCO_2_ condition. On the other hand, plant height (54.8 ± 1.0 cm), total tillers (17.7 ± 0.9), reproductive tillers (13.0 ± 0.5) and percent reproductive tillers (74.12 ± 2.1%) were recorded as the lowest in e0_3_. Under eT+eCO_2_, plant height (62.4 ± 0.6 cm) and total tillers (23.6 ± 0.6) were enhanced but reproductive tillers (18.6 ± 0.6) and percent reproductive tillers (78.8 ± 1.6%) were negatively affected as compared to ambient control (**Table 4**). Under *N. lugens* infestation, the plant parameters were recorded lower in all the treatments including ambient conditions. The plant height (63.1 ± 0.4 cm), total tillers (23.3 ± 0.4), reproductive tillers (18.9 ± 0.3) and percent reproductive tillers (81.2 ± 1.1%) were recorded maximum in eCO_2_ condition. On the other hand, plant height (51.6 ± 0.4 cm), total tillers (15.1 ± 0.8), reproductive tillers (10.1 ± 0.5) and percent reproductive tillers (67.1 ± 2.4%) were recorded as the lowest in e0_3_ infested with *N. lugens* among all the treatments (**Table 4**). Similarly, grain and yield traits significantly differed among the treatments in both uninfested, and infested conditions. The eCO_2_ has a positive effect on the length of the panicle (27.5 ± 0.3 cm), grain per panicle (99.7 ± 1.9), grain yield (40.1 ± 0.3 gm/hill), 1000 seed weight (40.1 ± 0.3 gm) as well as decrease the unfilled grain percentage (5.2 ± 0.5%) (**Table 5**). However, grains per panicle drastically decreased in *N. lugens* infestation (86.0 ± 2.8) under eCO_2_. The e0_3_ negatively hampered grain parameters like grains per panicle (74.7 ± 1.5), grain yield (27.5 ± 0.2 gm/hill) and 1000 seed weight (18.3 ± 0.4 gm). Also, the percentage of unfilled grain (12.2 ± 0.8%) under e0_3_ was the highest among all treatments. The length of the panicle under eT+eCO_2_ (26.8 ± 0.2 cm) increased significantly in uninfested conditions but it was at par with ambient control in infested conditions (**Table 5**).

**Table 4:** Rice growth and developmental parameters in Free Air Temperature Enrichment (FATE).

Treatment	Plant parameters*							
	Uninfested				Infested			
	Plant height (cm)	Total tillers (no.)	Reproductive tillers (no.)	Percent reproductive tillers	Plant height (cm)	Total tillers (no.)	Reproductive tillers (no.)	Percent reproductive tillers
eCO_2_	65.4 ± 0.8^a^	25.0 ± 0.8^a^	22.2 ± 0.6^a^	88.9 ± 1.0^a^	63.1 ± 0.4^a^	23.3 ± 0.4^a^	18.9 ± 0.3^a^	81.2 ± 1.1^a^
eT+eCO_2_	62.4 ± 0.6^b^	23.6 ± 0.6^a^	18.6 ± 0.6^b^	78.8 ± 1.6^b^	60.0 ± 0.8^b^	23.0 ± 0.6^a^	17.4 ± 0.7^a^	75.5 ± 2.1^b^
e0_3_	54.8 ± 1.0^c^	17.7 ± 0.9^c^	13.0 ± 0.5^c^	74.1 ± 2.1^c^	51.6 ± 0.4^c^	15.1 ± 0.8^c^	10.1 ± 0.5^c^	67.1 ± 2.4^c^
AM	60.9 ± 0.6^b^	22.2 ± 0.9^b^	19.1 ± 0.9^b^	85.8 ± 1.6^a^	58.3 ± 0.7^b^	20.0 ± 0.7^b^	16.0 ± 0.8^b^	79.6 ± 1.5^a^
***F- value***	28.5	14.3	29.2	16.6	56.8	33.2	37.1	11.1
***P – value***	<0.001	<0.001	<0.001	<0.001	<0.001	<0.001	<0.001	<0.001

*Mean of ten replications; Numbers with the same superscripts do not differ significantly.

F-value, test statistic from ANOVA indicating variance among treatments; p-value, probability value indicating statistical significance of differences among treatments (values <0.001 denote highly significant differences).

**Table 5:** Rice yield parameters in Free Air Temperature Enrichment (FATE).

Treatment	Grain and yield parameters*										
	Un-infested					Infested					
	Length of panicle (cm)	Grains/ panicle	Unfilled grains/ Panicle (%)	Grain yield/hill (g)	1000 seed weight (g)	Length of panicle (cm)	Grains/ Panicle	Unfilled grains/ Panicle (%)	Grain yield/hill (g)	1000 seed weight	% Yield reduction over uninfested
eCO_2_	27.5 ± 0.3^a^	99.7 ± 1.9^a^	5.2 ± 0.5^c^	40.1 ± 0.3^a^	22.8 ± 0.4^a^	25.3 ± 0.5^a^	86.0 ± 2.8^a^	5.6 ± 0.6^b^	33.7 ± 0.6^a^	17.7 ± 0.2^a^	15.9
eT+eCO_2_	26.8 ± 0.2^a^	89.9 ± 2.1^b^	7.1 ± 0.4^b^	38.1 ± 0.4^b^	20.4 ± 0.3^b^	24.2 ± 0.6^b^	83.0 ± 2.3^a^	7.2 ± 0.7^b^	33.7 ± 0.5^a^	17.0 ± 0.2^a^	11.5
e0_3_	20.2 ± 0.8^c^	74.7 ± 1.5^d^	12.2 ± 0.8^a^	27.5 ± 0.2^d^	18.3 ± 0.4^c^	18.0 ± 1.0^c^	69.6 ± 3.2^b^	13.6 ± 0.8^a^	25.1 ± 0.5^b^	15.6 ± 0.3^b^	8.72
AM	25.4 ± 0.6^b^	83.4 ± 1.8^c^	8.4 ± 0.5^b^	35.8 ± 0.4^c^	20.4 ± 0.4^b^	23.6 ± 0.6^b^	80.9 ± 1.4^a^	7.3 ± 0.6^b^	34.0 ± 0.6^a^	17.6 ± 0.2^a^	5.0
***F-value***	31.6	31.0	21.8	200.5	20.0	18.4	7.9	24.1	52.7	10.0	–
***P-value***	<0.001	<0.001	<0.001	<0.001	<0.001	<0.001	<0.001	<0.001	<0.001	<0.001	

*Mean of ten replications; Numbers with the same superscripts do not differ significantly.

F-value, test statistic from ANOVA indicating variance among treatments; p-value, probability value indicating statistical significance of differences among treatments (values <0.001 denote highly significant differences).

## 4 Discussion

### 4.1 Impact of climate change parameters on population dynamics of *N. lugens*

To effectively manage *N. lugens* in changing climatic scenarios, it is necessary to comprehend the incidence and dynamics of pest populations. The present research found that the population of *N. lugens*, which includes nymphs and brachypterous females, was maximum under eCO_2_ condition. The population of *N. lugens* reached its highest peak in early season, and a second peak was seen later in the season. This is in line with Pandi et al., 2018a; Daravath et al., 2018; and Tenguri et al., 2023, who suggested that eCO_2_ concentration increased photosynthesis, plant canopy size, and tillering, creating an ideal environment for *N. lugens* and faster population growth. The eT+eCO_2_ had a higher *N. lugens* population than ambient but lower than eCO_2_ conditions due to the simultaneous increase in atmospheric CO_2_ and temperature, which may affect insect-plant interaction directly and indirectly (Shi et al., 2014). The combined effect of eT+eCO_2_ boosts the population of *N. lugens*, but not to the same extent as eCO_2_ alone, because, in addition to the effect of CO_2_, increased temperature causes decrease in stem water levels, affecting the population of BPH that feeds on phloem Shi et al. (2014). On the other hand, e0_3_ impeded the population expansion of *N. lugens*. The population’s growth may have been inhibited by a decrease in feeding rate, lower fertility, and a reduction in the number of females as a result of e0_3_. Our findings match with the results of previous studies (Walling, 2000; Yan et al., 2018) who suggested the elevated e0_3_ conditions altered the plant nutritional profile, synthesis and accumulate more secondary metabolite. Menendez et al. (2010) found a similar finding for the green peach aphid *M. persicae*. The impact of elevated ozone on insect development appears to be variable across different species. Some studies have reported that increased ozone levels enhance insect growth, while others have found a negative or negligible effect (Heliovaara and Vaisanen, 1993; Guo et al., 2020). For instance, in *Bemisia tabaci* (Q biotype), Hong et al. (2016) observed that elevated ozone led to increased egg production, shorter development time, and higher survivorship. Conversely, Mina et al. (2012) reported that elevated ozone had a detrimental effect on the development of *Chilo partellus.* In eCO_2_ and eT+eCO_2_, fecundity and egg masses per female were increased. Despite this, fecundity was greater in eCO_2_ conditions than in eT+eCO_2_. Elevated CO_2_ levels led to a greater population of *N. lugens*, indicating enhanced fecundity and a higher proportion of brachypterous females. This trend might be due to favorable microclimatic circumstances caused by dense plant growth and enhanced tillering (Prasannakumar et al., 2012). Elevated CO_2_ levels increased the number of brachypterous females, possibly increasing to the BPH population. These females produced eggs at a higher rate. Similarly, Krishnaiah et al. (2008) discovered that brachypterous females produce more eggs and contribute more to population growth than macropterous females. The eT+eCO_2_ increased fertility in rice plant hoppers, *N. lugens* (Pandi et al., 2018a), and maize leaf aphids, *R. maidis* (Xie et al., 2014), indicating a positive effect on multiplication. Plant nutritional quality, favorable microclimatic conditions, improved feeding, and a greater sucking rate by females may have contributed to higher fecundity. In contrast BPH strongly avoided rice plants that had been subjected to elevated ozone while choosing sites to lay eggs. This is most likely due to the changes in the chemical signals given by the plant, which are commonly employed to decide whether a host plant is accepted or not for feeding and egg laying (Hilker and Meiners, 2011). Our findings are consistent with prior study done by Cui et al., 2019 and Inoue et al., 2016. The studies also found that higher ozone levels affected fertility in the whitefly *B. tabaci* and fewer eggs were deposited by the leaf beetle *A. Coerulea*.

In eCO_2_ and eT+eCO_2_, the pest development period, including nymphal length and adult lifespan significantly decreased. The shortened developmental time may be attributed to the increased C:N ratio, higher sugar, and reduced nitrogen levels in the rice plants (SChadler et al., 2007). As a result, insects expended additional energy on feeding to make up the dietary deficiency of their food which ultimately shortened developmental period. Some recent researches have indicated that *N. lugens* on rice under CO_2_ enriched settings had shorter developmental period, shortened nymphal period, and short female and male lifespan (Pandi et al., 2018b; Daravath et al., 2018). In a study conducted by Auad et al. (2012), it was shown that the nymphal longevity of the yellow sugarcane aphid, *S. flava*, was dramatically reduced under elevated CO_2_+tempreture. Similarly, Shi et al. (2014) and Xie et al. (2014) found that female *N. lugens* had a shorter lifespan and had substantially shorter developmental durations at each life stage when exposed to elevated CO_2_ and temperature. However, elevated ozone extended nymphal and total developmental period of *N. lugens*. In both seasons, elevated ozone levels reduced adult lifetime, as well as female and male longevity. The elevated ozone level stimulated the plants to produce and store secondary metabolites. This might impact several facets of insect behavior and performance, such as feeding patterns, ability to lay eggs, longevity, and reproductive potential. These modifications have the capacity to modify the abundance and structure of herbivorous insects (Lindroth, 2010; Couture and Lindroth, 2012; Cui et al., 2012, 2014).

Honeydew production by insects is directly proportional to the sap sucking (Tenguri et al., 2023). The *N. lugens* reared under eCO_2_, and eT+eCO_2_ produced considerably more honeydew than AM. It shows that *N. lugens* females are sucking more to compensate for their inferior nutritional condition and the plant’s greater C: N ratio. Prior research has indicated that *N. lugens* exhibited an increased rate of sap-sucking under increasing CO_2_, as earlier reported by Shi et al. (2014); Pandi et al. (2018b), and Daravath et al. (2018). In contrast, significant lower honeydew production was reported by females fed on plants raised under elevated ozone conditions. Walling (2000) and Yan et al. (2018) observed that when plants are grown under elevated ozone concentrations, they produce more secondary metabolites and anti-nutritional chemicals, which reduce the female sap sucking rate.

### 4.2 Impact of climate change parameters on plant growth and yield attributes of rice

Agricultural crops are heavily influenced by variations in climatic circumstances such as CO_2_ levels, temperature, and ozone. Elevated CO_2_ has a nutritional and fertilization effect on plant growth and reproduction fertilization effect on plant growth and reproduction. This, in the end, results in higher biomass and productivity specifically in C_3_ plants (Hasegawa et al., 2007; Ainsworth et al., 2007; Reddy et al., 2010). Increasing CO_2_ concentrations will enhance the degree of damage caused by insect pests, in addition to promoting the growth of plants (Gregory et al., 2009). In this study, both eCO_2_ and the combination of eT+eCO_2_ had a positive effect on rice growth indices, such as plant height and the number of tillers. Similar impact is observed on grain characteristics such as panicle length, grain per panicle, test weight, and yield in uninfested rice crops. *N. lugens* infestation in rice plants causes more severe damage, notwithstanding the advantageous impact, when plants are subjected to elevated CO_2_ levels and the combined effect of elevated temperature and CO_2_. The increased level of damage was attributed to their higher fecundity, an increased number of wingless females, and intensified sap-sucking behavior (Prasannakumar et al., 2012). Shi et al. (2014) found that the combination of eT+eCO_2_ had a favorable effect on various biological parameters of both the rice plant and the plant hopper. Thus, *N. lugens* population significantly increases under eCO_2_ alone as well as its combination eT+eCO_2_, thereby increasing yield loss. In contrast, ozone impedes several reproductive processes, including germination of pollen, fertilization, and the abortion of flowers, pods, and individual ovules or seeds (Black et al., 2000), and reduces grain yield, straw yield and harvest index (Bhatia et al., 2012). Elevated ozone had a detrimental impact on plant development and yield characteristics, as plant height, total tillers and reproductive tillers, panicle length, and seeds per panicle were all considerably lower than under ambient condition. Negative responses of rice to elevated ozone have been attributed to impaired growth, photosynthetic performance, reproduction and quality of the grain ultimately showing a reduction in grain yield (Bhatia et al., 2021; Oksanen et al., 2013).

Overall, the findings demonstrated that when rice plants are exposed to elevated CO_2_, their nutritional, biochemical, and developmental properties improve. As a result, *N. lugens* grows and develops more effectively. The interactive effect of elevated CO_2_+temperature had a favorable impact on the growth and development of *N. lugens*, while the higher temperature may have counteracted and diminished the amplified favorable effect of elevated CO_2_. On the other hand, elevated ozone levels decreased plant nutrition by interfering with certain plant growth factors. Hence, the number of *N. lugens* decreased and its peak was delayed than expected.

## 5 Conclusion

In conclusion, the study revealed that eCO_2_ alone had a positive effect on rice plant growth and yield parameters but simultaneously also stimulated the *N. lugens* population development. The surge in *N. lugens* population might be due to the formation of a favorable micro-climate by denser plant growth which resulted in a higher number of brachypterous females and nymph population, and also higher fecundity by females. This increased brown plant hopper development coupled with higher sap sucking rate under enriched CO_2_ resulted in greater yield losses compared to ambient conditions. Similarly, the interactive effect of elevated temperature and CO_2_ also had certain positive effects on rice plant growth, reproductive and grain parameters. However, it favored the pest multiplication and perpetuation causing higher grain yield losses than ambient conditions. Also, the elevated ozone concentration above threshold level had a significant negative effect on rice plant growth and yield, this negative effect of Ozone accompanied with *N. lugens* infestation aggravates more yield losses than ambient conditions. The *N. lugens* populations are expected to aggravate in future climate change conditions.

## Data Availability

The original contributions presented in the study are included in the article/**Supplementary Material**. Further inquiries can be directed to the corresponding author.

## References

[B1] Ainsworth E. A. Rogers A. Leakey A. D. Heady L. E. Gibon Y. Stitt M.. (2007). Does elevated atmospheric [CO_2_] alter diurnal C uptake and the balance of C and N metabolites in growing and fully expanded soybean leaves? J. Exp. Botany. 58, 579–591. doi: 10.1093/jxb/erl233 17158509

[B2] Auad A. M. Fonseca M. G. Resende T. T. Maddalena I. S. C. P. (2012). Effect of climate change on longevity and reproduction of *Sipha flava* (Hemiptera: Aphididae). Florida Entomologist. 95, 433–444. doi: 10.2307/23268565

[B3] Babu S. B. Parameswaran C. Anant A. K. Padhi J. Bansal R. Priyadarsini S.. (2022). Genomic analysis and finding of candidate genes for *Nilaparvata lugens* (stål) resistance in Indian pigmented and other indigenous rice genotypes. Crop Protection. 156, 105959. doi: 10.1016/j.cropro.2022.105959

[B4] Bale J. S. Masters G. J. Hodkinson I. D. Awmack C. Bezemer T. M. Brown V. K. (2002). Herbivory in global climate change research: direct effects of rising temperature on insect herbivores. Glob Chang Biol. 8, 1–16. doi: 10.1046/j.1365-2486.2002.00451.x

[B5] Behura N. Sen P. Kar M. K. (2011). Introgression of yellow stem borer (*Scirphophaga oryzae*) resistance gene, into cultivated rice (*Oryza* sp.) from wild spp. Indian J. Agric. Sci. 81, 359–362.

[B6] Bhatia A. Mina U. Kumar V. Tomer R. Kumar A. Chakrabarti B.. (2021). Effect of elevated ozone and carbon dioxide interaction on growth, yield, nutrient content and wilt disease severity in chickpea grown in Northern India. Heliyon. 7, e06049. doi: 10.1016/j.heliyon.2021.e06049 33537483 PMC7841360

[B7] Bhatia A. Tomer R. Kumar V. Singh S. D. Pathak H. (2012). Impact of tropospheric ozone on crop growth and productivity–a review. J. scient. Ind. Res. 71, 97–112.

[B8] Black V. J. Black C. R. Roberts J. A. Stewart C. A. (2000). Impact of ozone on the reproductive development of plants. New Phytologist. 147, 421–447. doi: 10.1046/j.1469-8137.2000.00721.x 33862931

[B9] Brauer M. Freedman G. Frostad J. Van Donkelaar A. Martin R. V. Dentener F.. (2016). Ambient air pollution exposure estimation for the global burden of disease 2013. Environ. Sci. Technol. 50, 79–88. doi: 10.1021/acs.est.5b03709 26595236

[B10] Brownlie J. C. Johnson K. N. (2009). Symbiont-mediated protection in insect hosts. Trends Microbiol. 17, 348–354. doi: 10.1016/j.tim.2009.05.005 19660955

[B11] Capone A. Ricci I. Damiani C. Mosca M. Rossi P. Scuppa P.. (2013). Interactions between Asaia, Plasmodium and Anopheles: new insights into mosquito symbiosis and implications in malaria symbiotic control. Parasit Vectors. 6, 182. doi: 10.1186/1756-3305-6-182 23777746 PMC3708832

[B12] Chen F. J. Wu G. Ge F. (2004). Impacts of elevated CO_2_ on the population abundance and reproductive activity of aphid Sitobion avenae Fabricius feeding on spring wheat. J. Appl. Entomol. 128, 723–730. doi: 10.1111/j.1439-0418.2004.00921.x

[B13] Cheng J. Zhao W. Lou Y. Zhu Z. (2001). Intra- and inter-specific effects of the brown planthopper and white backed planthopper on their population performance. J. Asia-Pac. Entomol. 4, 85–92. doi: 10.1016/S1226-8615(08)60108-9

[B14] Coakley S. M. Scherm H. Chakraborty S. (1999). Climate change and disease management. Annu. Rev. Phytopathol. 37, 399–426. doi: 10.1146/annurev.phyto.37.1.399 11701829

[B15] Coley P. Massa M. Lovelock C. Winter K. (2002). Effects of elevated CO_2_ on foliar chemistry of saplings of nine species of tropical tree. Oecologia. 133, 62–69. doi: 10.1007/s00442-002-1005-6 24599370

[B16] Couture J. Lindroth R. L. (2012). Atmospheric change alters performance of an invasive forest insect. Glob Chang Biol. 18, 3543–3557. doi: 10.1111/gcb.12014

[B17] Cui H. Sun Y. Sun J. Ren Q. Li C. Ge F. (2012). Elevated O_3_ reduces the fitness of *Bemisia tabaci* via enhancement of the SA-dependent defense of the tomato plant. Arthropod-Plant Interact. 6, 425–437. doi: 10.1007/s11829-012-9189-0

[B18] Cui H. Sun J. Wei Y. Hu F. Ge F. (2014). Elevated O_3_ enhances the attraction of whitefly infested tomato plants to *Encarsia formosa* . Sci. Rep. 4, 1–6. doi: 10.1038/srep05350 PMC4061550 24939561

[B19] Cui H. Sun Y. Zhao Z. Zhang Y. (2019). The combined effect of elevated O_3_ levels and TYLCV infection increases the fitness of *Bemisia tabaci* Mediterranean on tomato plants. Environ. Entomol. 48, 1425–1433. doi: 10.1093/ee/nvz113 31586399 PMC6885742

[B20] Daravath V. Chander S. Mandla R. (2018). Impact of elevated CO_2_ on *Nilaparvata lugens* (stal), rice crop and feeding of *Pardosa pseudoannulata* . Indian J. Entomol. 80, 662–667. doi: 10.5958/0974-8172.2018.00220.1

[B21] Gregory P. J. Johnson S. N. Newton A. C. Ingram J. S. I. (2009). Integrating pests and pathogens into the climate change/food security debate. J. Exp. Bot. 60, 2827–2838. doi: 10.1093/jxb/erp080 19380424

[B22] Guo H. Sun Y. Yan H. Li C. Ge F. (2020). O_3_-induced priming défense associated with the abscisic acid signaling pathway enhances plant resistance to *bemisia tabaci* . Front. Plant Sci. 11. doi: 10.3389/fpls.2020.00093 PMC7069499 32210979

[B23] Hasegawa T. Shimono H. Yang L. X. Kim H. Y. Kobayashi T. Sakai H.. (2007). “Response of rice to increasing CO_2_ and temperature: recent findings from large-scale free-air CO_2_ enrichment (FACE) experiments,” in Proceedings of the 26th international rice conference, 9–12 Octobe. Eds. Aggarwal P. Ladha J. Singh R. Devakumar C. Hardy B. (New Delhi, India: International Rice Research Institute, Indian Council of Agricultural Research and National Academy of Agricultural Sciences), 439–447.

[B24] Heliovaara K. Vaisanen R. (1993). “Pollution in terrestrial ecosystems,” in Insects and Pollution (CRC Press, Boca Raton, Florida), 55–160.

[B25] Hilker M. Meiners T. (2011). Plants and insect eggs: how do they affect each other? Phytochemistry. 72, 1612–1623. doi: 10.1016/j.phytochem.2011.02.018 21439598

[B26] Hong Y. Yi T. Tan X. Zhao Z. Ge F. (2016). High ozone (O_3_) affects the fitness associated with the microbial composition and abundance of Q biotype. Bemisia tabaci. Front. Microbiol. 7. doi: 10.3389/fmicb.2016.01593 PMC5065991 27799921

[B27] Horgan F. G. Arida A. Ardestani G. Almazan M. L. P. (2007). Elevated temperatures diminish the effects of a highly resistant rice variety on the brown planthopper. Sci. Rep. 11(1), 262. doi: 10.1038/s41598-020-80704-4 PMC7794346 33420350

[B28] Inoue W. A. Vanderstock T. Sakikawa M. Nakamura H. Saito H. Shibuya M.. (2016). The interaction between insects and deciduous broadleaved trees under different O_3_ concentrations and soil fertilities. Boreal For. Res. 64, 30.

[B29] IPCC (2022). “Summary for Policymakers,” in Climate Change 2022: Impacts, Adaptation, and Vulnerability. Contribution of Working Group II to the Sixth Assessment Report of the Intergovernmental Panel on Climate Change (Cambridge University Press, Cambridge, UK and New York, NY, USA), 3–33. doi: 10.1017/9781009325844.001

[B30] Khush G. S. (2004). Harnessing science and technology for sustainable rice-based production systems. Int. Rice Commission Newslett. 53, 17–23.

[B31] Krishnaiah N. V. Lekshmi V. J. Passlu I. C. Kalti G. R. Padmavathi C. (2008). Insecticide in rice-IPM, past, present and future, DRR, hyderabad, India 148.

[B32] Lindroth R. L. (2010). Impacts of elevated atmospheric CO_2_ and O_3_ on forests: Phytochemistry, trophic interactions, and ecosystem dynamics. J. Chem. Ecol. 36, 2–21. doi: 10.1007/s10886-009-9731-4 20054619

[B33] Long S. P. (2012). Virtual special issue on food security—greater than anticipated impacts of near-term global atmospheric change on rice and wheat. Glob Chang Biol. 18, 1489–1490. doi: 10.1111/j.1365-2486.2012.02676.x

[B34] Menendez A. I. Romero A. M. Folcia A. M. Martinez-Ghersaet M. A. (2010). Aphid and episodic O_3_ injury in arugula plants (*Eruca sativa* Mill.) grown in open-top field chambers. Agric. Ecosyst. Environ. 135, 10–14. doi: 10.1016/j.agee.2009.08.005

[B35] Mina U. Bhatia A. Chakrabarti B. Harit R. C. Kumar U. (2012). Impact of ozone and carbon dioxide on growth and development of *Chilo partellus* swinhoe (maize stalk borer). J. Entomol. Res. 36, 363–366.

[B36] Morgan P. B. Mies T. A. Bollero A. Nelson R. L. Long S. P. (2006). Season-long elevation of ozone concentration to projected 2050 levels under fully open-air conditions substantially decreases the growth and production of soybean. New Phytol. 170, 333–343. doi: 10.1111/j.1469-8137.2006.01679.x 16608458

[B37] Oksanen E. Pandey V. Pandey A. K. Keski-Saari S. Kontunen-Soppela S. Sharma S. (2013). Impacts of increasing ozone on Indian plants. Environ. Pollut. 177, 189–200. doi: 10.1016/j.envpol.2013.02.010 23466168

[B38] Paguia P. Pathak M. D. Heinrichs E. A. (1980). Honeydew excretion measurement techniques for determining differential feeding activity of biotype of *Nilaparvata lugens* on rice varieties. J. Econ. Entomol. 73, 35–40. doi: 10.1093/jee/73.1.35

[B39] Pandi G. G. P. Chander S. Pal M. Soumia P. S. (2018b). Impact of elevated CO_2_ on *Oryza sativa* phenology and brown planthopper, *Nilaparvata lugens* (Hemiptera: Delphacidae) population. Curr. Sci. 114, 1767–1777. doi: 10.18520/cs/v114/i08/1767-1777

[B40] Pandi G. G. P. Chander S. Singh M. P. Pathak H. (2018a). Impact of elevated CO_2_ and temperature on brown planthopper population in rice ecosystem. Proc. Natl. Acad. Sci. India Sect B Biol. Sci. 88, 57–64. doi: 10.1007/s40011-016-0727-x PMC5846970 29568154

[B41] Prasannakumar N. Chander S. Pal M. (2012). Assessment of impact of climate change with reference to elevated CO_2_ on rice brown planthopper, *Nilaparvata lugens* (Stal.) and crop yield. Curr. Sci. 103, 1201–1205.

[B42] Raderschall C. Vico G. Lundin O. Taylor A. Bommarco R. (2021). Water stress and insect herbivory interactively reduce crop yield while the insect pollination benefit is conserved. Glob Chang Biol. 27, 1–13. doi: 10.1111/gcb.15386 33118276 PMC7756552

[B43] Reddy A. R. Rasineni G. K. Raghavendra A. S. (2010). The impact of global elevated CO_2_ concentration on photosynthesis and plant productivity. Curr. Sci. 99, 46–57.

[B44] SChadler M. Roeder M. Brandal R. Matthies D. (2007). Interacting effects of elevated CO_2_, nutrient availability and plant species on a generalist invertebrate herbivore. Glob Chang Biol. 13, 1005–1015. doi: 10.1111/j.1365-2486.2007.01319.x

[B45] Shi B. K. Huang J. L. Hu C. X. Hou M. L. (2014). Interactive effects of elevated CO_2_ and temperature on rice planthopper, *Nilaparvata lugens* . J. Integr. Agric. 13, 1520–1529. doi: 10.1016/S2095-3119(14)60804-2

[B46] Srinivasa Rao M. Srinivas K. Vanaja M. Rao G. S. N. Venkateswarlu B. Ramakrishna Y. S. (2009). Host plant (Ricinus communis Linn.) mediated effects of elevated CO2 on growth performance of two insect folivores. Curr. Sci. 97, 1047–1054.

[B47] Tenguri P. Chander S. Ellur R. K. Yele Y. Sundaran A. P. Nagaraju M. T.. (2023). Effect of silicon application to the rice plants on feeding behavior of the brown planthopper, *nilaparvata lugens* (Stål) under elevated CO_2_ . Silicon. 15, 5811–5820. doi: 10.1007/s12633-023-02480-w

[B48] Walling L. L. (2000). The myriad plant responses to herbivores. J. Plant Growth Regul. 19, 195–216. doi: 10.1007/s003440000026 11038228

[B49] Wang J. J. Tsai J. H. (2001). Development, survival and reproduction of black citrus aphid, Toxoptera aurantii (Hemiptera: Aphididae), as a function of temperature. Bull. Entomol. Res. 91, 477–487. doi: 10.1079/BER2001120 11818043

[B50] Ward N. L. Masters G. L. (2007). Linking climate change and species invasion: an illustration using insect herbivores. Glob Chang Biol. 13, 1605–1615. doi: 10.1111/j.1365-2486.2007.01399.x

[B51] Way D. A. Oren R. (2010). Differential responses to changes in growth temperature between trees from different functional groups and biomes: a review and synthesis of data. Tree Physiol. 30, 669–688. doi: 10.1093/treephys/tpq015 20368338

[B52] Xie H. Zhao L. Wang W. Wang Z. Ni X. Cai W.. (2014). Changes in life history parameters of *Rhopalosiphum maidis* (Homoptera: Aphididae) under four different elevated temperature and CO_2_ combinations. J. Econ. Entomol. 107, 1411–1418. doi: 10.1603/EC13302 25195429

[B53] Yan H. Guo H. Yuan E. Su Y. Ge F. (2018). Elevated CO_2_ and O_3_ alter the feeding efficiency of *Acyrthosiphon pisum* and *Aphis craccivora* via changes in foliar secondary metabolites. Sci. Rep. 8, 996. doi: 10.1038/s41598-018-28020-w 29967388 PMC6028383

