# Supplementary material for: “Physiological and demographic responses of Nilaparvata lugens to combined climate stressors: CO2, temperature, and ozone”
Source: Front Plant Sci. 2025 Jun 5;16:1518361. doi: 10.3389/fpls.2025.1518361 (PMC12176854; doi:10.3389/fpls.2025.1518361)

**Table 1. *N. lugens* population/hill (nymphs, females and males) in FATE during 2019**

|  | ***N. lugens* population (Nymph, Male & Female) *** | | | | | | | | | |
| --- | --- | --- | --- | --- | --- | --- | --- | --- | --- | --- |
|  | **Weeks after adult release (WAR)** | | | | | | | | | |
| **Treatments** | **1** | **2** | **3** | **4** | **5** | **6** | **7** | **8** | **9** | **Mean±SE** |
| **eT+eCO_2_** | 2.9±0.5 | 12.2±0.6 | 29.7±2.6 | 47.8±2.3 | 66.0±4.6 | 65.8±3.4 | 60.9±2.1 | 87.7±4.3 | 28.1±2.2 | 44.5±9.4 |
|  | (1.9±0.1) | (3.6±0.1) | (5.4±0.2) | (6.9±0.2) | (8.1±0.3) | (8.1±0.2) | (7.8±0.1) | (9.3±0.2) | (5.3±0.2) | (6.3±0.8)^b^ |
| **eCO_2_** | 4.5±0.3 | 13.5±0.9 | 30.7±1.9 | 73.9±5.1 | 114.6±6.2 | 99.2±7.4 | 78.2±7.1 | 100.7±5.6 | 39.5±2.9 | 61.6±13.5 |
|  | (2.3±0.1) | (3.7±0.1) | (5.6±0.2) | (8.6±0.3) | (10.7±0.3) | (9.9±0.4) | (8.8±0.4) | (10.0±0.3) | (6.3±0.2) | (7.3±1.0)^a^ |
| **eO_3_** | 3.4±0.5 | 6.2±0.6 | 17.6±1.3 | 20.9±1.7 | 26.4±2.2 | 28.2±2.3 | 21.8±2.0 | 26.4±2.2 | 8.8±1.5 | 17.7±3.1 |
|  | (2.0±0.1) | (2.6±0.1) | (4.2±0.2) | (4.6±0.2) | (5.1±0.2) | (5.3±0.2) | (4.7±0.2) | (5.1±0.2) | (3.0±0.3) | (4.1±0.4)^d^ |
| **AM (Ambient)** | 4.9±0.5 | 6.8±0.7 | 16.6±1.6 | 29.2±2.7 | 41.2±4.4 | 35.9±2.7 | 28.6±2.5 | 40.9±4.3 | 14.6±1.6 | 24.3±4.7 |
|  | (2.4±0.1) | (2.7±0.1) | (4.1±0.2) | (5.4±0.3) | (6.4±0.3) | (6.0±0.2) | (5.3±0.2) | (6.3±0.3) | (3.9±0.2) | (4.8±0.5)^c^ |
| **Mean±SE** | 3.9±0.5 | 9.7±1.9 | 23.7±3.8 | 43.0±11.8 | 62.1±19.3 | 57.3±16.2 | 47.3±13.4 | 63.9±17.9 | 22.7±6.9 |  |
|  | (2.2±0.1)^f^ | (3.2±0.3)^e^ | (4.9±0.4)^d^ | (6.4±0.9)^c^ | (7.6±1.2)^a^ | (7.3±1.0)^ab^ | (6.7±1.0)^c^ | (7.7±1.2)^a^ | (4.6±0.7)^d^ |  |

Treatments, F= (373.5), LSD= (0.21), P<0.001

Weeks, F= (313.6), LSD= (0.31), P<0.001

Interactions, F= (14.8), LSD= (0.63), P<0.001

Planthopper count with different subscript differ significantly

*Average of ten replication

Numbers in parenthesis are SQRT (X+1) valued

**Table 2. *N. lugens* nymphal population/hill in FATE during 2019**

|  | **Nymph population*** | | | | | | | | |  |
| --- | --- | --- | --- | --- | --- | --- | --- | --- | --- | --- |
|  | **Weeks after adult release (WAR)** | | | | | | | | |  |
| **Treatments** | **1** | **2** | **3** | **4** | **5** | **6** | **7** | **8** | **9** | **Mean±SE** |
| **eT+eCO_2_** | 1.9±0.4 | 7.6±0.5 | 21.1±2.2 | 32.9±1.9 | 47.3±4.1 | 46.2±3.6 | 37.1±2.7 | 58.1±3.6 | 14.7±1.6 | 29.6±6.4 |
|  | (1.6±0.1) | (2.9±0.1) | (4.6±0.2) | (5.8±0.2) | (6.8±0.3) | (6.5±0.3) | (6.1±0.2) | (7.6±0.2) | (3.8±0.2) | (5.2±0.6)^b^ |
| **eCO_2_** | 3.6±0.3 | 5.3±0.4 | 19.7±1.4 | 52.9±5.2 | 86.2±4.8 | 78.9±6.9 | 59.1±6.2 | 67.1±4.2 | 14.4±2.2 | 43.0±10.8 |
|  | (2.1±0.1) | (2.4±0.1) | (4.5±0.2) | (7.2±0.4) | (9.3±0.3) | (8.8±0.4) | (7.6±0.4) | (8.2±0.3) | (3.8±0.3) | (6.0±0.9)^a^ |
| **eO_3_** | 3.4±0.5 | 5.1±0.5 | 12.4±1.1 | 15.5±1.5 | 13.6±1.6 | 18.3±2.7 | 15.4±1.1 | 13.6±1.6 | 4.7±1.1 | 11.3±1.8 |
|  | (2.0±0.1) | (2.4±0.1) | (3.6±0.2) | (4.0±0.2) | (3.7±0.2) | (4.2±0.3) | (4.0±0.1) | (3.7±0.2) | (2.2±03) | (3.4±0.2)^d^ |
| **AM (Ambient)** | 3.4±0.4 | 3.8±0.5 | 9.7±1.1 | 22.1±2.6 | 26.4±3.7 | 25.9±2.0 | 23.3±2.2 | 26.4±3.7 | 6.3±1.6 | 16.4±3.3 |
|  | (2.3±0.1) | (2.1±0.1) | (3.2±0.2) | (4.7±0.3) | (5.1±0.3) | (5.1±0.2) | (4.8±0.2) | (5.1±0.3) | (2.5±0.3) | (3.9±0.4)^c^ |
| **Mean±SE** | 3.3±0.5 | 5.4±0.7 | 15.7±2.7 | 30.8±8.1 | 43.3±15.8 | 42.3±13.5 | 33.7±9.5 | 41.3±12.7 | 10.0±2.6 |  |
|  | (2.06±0.1)^f^ | (2.5±0.1)^e^ | (4.0±0.3)^c^ | (5.5±0.7)^b^ | (6.3±1.1)^a^ | (6.3±1.0)^a^ | (5.7±0.8)^b^ | (6.2±1.0)^a^ | (3.2±0.4)^d^ |  |

Treatments, F= (229.9), LSD= (0.22), P<0.001

Weeks, F= (206.3), LSD= (0.33), P<0.001

Interactions, F= (15.3), LSD= (0.66), P<0.001

Planthopper count with different subscript differ significantly

*Average of ten replication

Numbers in parenthesis are SQRT (X+1) valued

**Table 3. *N. lugens* female Population/hill in FATE during 2019**

|  | **Female population*** | | | | | | | | |  |
| --- | --- | --- | --- | --- | --- | --- | --- | --- | --- | --- |
|  | **Weeks after adult release (WAR)** | | | | | | | | |  |
| **Treatments** | **1** | **2** | **3** | **4** | **5** | **6** | **7** | **8** | **9** | **Mean±SE** |
| **eT+eCO_2_** | 0.0±0.0 | 2.0±0.3 | 4.9±0.6 | 10.3±1.0 | 13.6±1.1 | 14.8±1.2 | 14.6±1.6 | 18.4±1.6 | 4.2±0.9 | 9.2±2.1 |
|  | (1.0±0.0) | (1.7±0.1) | (2.3±01) | (3.3±0.1) | (3.7±0.14) | (3.9±0.14) | (3.9±0.2) | (4.3±0.17) | (2.2±0.18) | (2.9±0.39)^b^ |
| **eCO_2_** | 0.0±0.0 | 2.4±0.5 | 3.7±0.5 | 11.7±0.9 | 12.3±1.9 | 14.8±1.7 | 13.9±1.3 | 13.9±1.8 | 18.2±1.3 | 10.1±2.1 |
|  | (1.0±0.0) | (1.7±0.1) | (2.1±0.1) | (3.5±0.1) | (3.5±0.2) | (3.9±0.23) | (3.8±0.17) | (3.8±0.22) | (4.3±0.15) | (3.1±0.38)^a^ |
| **eO_3_** | 0.0±0.0 | 1.1±0.2 | 3.5±0.5 | 4.2±0.6 | 7.5±1.0 | 5.5±0.9 | 6.4±1.3 | 7.5±1.0 | 2.5±0.7 | 4.2±0.9 |
|  | (1.0±0.0) | (1.4±0.08) | (2.0±0.1) | (2.2±0.1) | (2.8±0.18) | (2.4±0.17) | (2.6±0.25) | (2.8±0.18) | (1.8±0.16) | (2.2±0.22)^c^ |
| **AM (Ambient)** | 0.0±0.0 | 1.9±0.3 | 3.8±0.6 | 4.6±1.0 | 7.7±1.1 | 7.9±1.0 | 2.7±0.7 | 7.7±1.1 | 5.9±1.4 | 4.6±0.9 |
|  | (1.0±0.0) | (1.6±0.1) | (2.1±0.1) | (2.2±0.2) | (2.8±0.19) | (2.9±0.17) | (1.8±0.18) | (2.8±0.19) | (2.4±0.28) | (2.2±0.22)^c^ |
| **Mean±SE** | 0.0±0.0 | 1.8±0.27 | 3.9±0.31 | 7.7±1.9 | 10.2±1.5 | 10.7±2.3 | 9.4±2.9 | 11.8±2.6 | 7.7±3.5 |  |
|  | (1.0±0.0)^f^ | (1.6±0.07)^e^ | (2.1±0.06)^d^ | (2.8±0.34)^c^ | (3.2±0.23)^a^ | (3.3±0.36)^a^ | (3.0±0.49)^b^ | (3.4±0.36)^a^ | (2.7±0.56)^c^ |  |

Treatments, F= (76.3), LSD= (0.15), P<0.001

Weeks, F= (101.4), LSD= (0.23), P<0.001

Interactions, F= (8.6), LSD= (0.46), P<0.001

Planthopper count with different subscript differ significantly

*Average of ten replication

Numbers in parenthesis are SQRT (X+1) valued

**Table 4. *N. lugens* male population/hill in FATE during 2019**

|  | **Male population*** | | | | | | | | | | |
| --- | --- | --- | --- | --- | --- | --- | --- | --- | --- | --- | --- |
|  | **Weeks after adult release (WAR)** | | | | | | | | | | |
| **Treatments** | **1** | **2** | **3** | **4** | **5** | **6** | **7** | **8** | **9** | **Mean±SE** |  |
| **eT+eCO_2_** | 1.0±0.2 | 2.6±0.3 | 3.7±0.8 | 4.6±0.8 | 5.1±0.8 | 4.8±0.7 | 9.2±0.9 | 11.2±1.2 | 10.4±1.1 | 5.8±1.2 |  |
|  | (1.3±0.1) | (1.8±0.1) | (2.0±0.2) | (2.2±0.2) | (2.4±0.2) | (2.3±0.2) | (3.1±0.1) | (3.4±0.2) | (3.3±0.2) | (2.4±0.2)^b^ |  |
| **eCO_2_** | 0.9±0.2 | 5.8±0.5 | 7.3±1.1 | 9.3±1.5 | 16.1±2.5 | 5.5±0.9 | 5.2±1.1 | 19.7±1.4 | 6.9±1.4 | 8.5±2.0 |  |
|  | (1.3±0.1) | (2.5±0.1) | (2.7±0.2) | (3.1±0.2) | (3.9±0.4) | (2.4±0.2) | (2.3±0.2) | (4.5±0.2) | (2.6±0.3) | (2.8±0.3)^a^ |  |
| **eO_3_** | 0.0±0.0 | 0.0±0.0 | 1.7±0.4 | 1.2±0.4 | 5.3±1.7 | 4.4±0.7 | 0.0±0.0 | 5.3±1.7 | 1.6±0.5 | 2.1±0.7 |  |
|  | (1.0±0.0) | (1.0±0.0) | (1.6±0.1) | (1.4±0.1) | (2.2±0.3) | (2.2±0.2) | (1±0.0) | (2.2±0.3) | (1.5±0.2) | (1.6±0.2)^d^ |  |
| **AM (Ambient)** | 0.5±0.2 | 1.1±0.2 | 3.1±0.5 | 2.5±0.5 | 7.1±0.7 | 2.1±0.8 | 2.6±0.6 | 6.8±0.7 | 2.4±0.6 | 3.1±0.8 |  |
|  | (1.2±0.1) | (1.4±0.1) | (1.9±0.1) | (1.8±0.2) | (2.8±0.1) | (1.6±0.2) | (1.8±0.2) | (2.7±0.1) | (1.7±0.2) | (1.9±0.2)^c^ |  |
| **Mean±SE** | 0.6±0.2 | 2.3±1.3 | 3.9±1.2 | 4.4±1.8 | 8.4±2.6 | 4.2±0.7 | 4.2±2.0 | 10.7±3.2 | 5.3±2.1 |  |  |
|  | (1.2±0.1)^f^ | (1.7±0.3)^e^ | (2.1±0.2)^d^ | (2.1±0.4)^d^ | (2.8±0.4)^b^ | (2.1±0.2)^d^ | (2.0±0.5)^d^ | (3.2±0.5)^a^ | (2.3±0.4)^c^ |  |  |

Treatments, F= (85.5), LSD= (0.17), P<0.001

Weeks, F= (40.1), LSD= (0.25), P<0.001

Interactions, F= (5.6), LSD= (0.51), P<0.001

Planthopper count with different subscript differ significantly

*Average of ten replication

Numbers in parenthesis are SQRT (X+1) valued

**Table 5. *N. lugens* population/hill (nymphs, males and females) in FATE during 2020**

|  | ***N. lugens* population (Nymph+Female+Male) *** | | | | | | |
| --- | --- | --- | --- | --- | --- | --- | --- |
|  | **Weeks after adult release (WAR)** | | | | | | |
| **Treatments** | **1** | **2** | **3** | **4** | **5** | **6** | **Mean±SE** |
| **eT+eCO_2_** | 4.5±0.6 | 24.3±2.3 | 72.4±5.3 | 75.9±3.8 | 70.5±4.4 | 37.9±1.6 | 47.5±12.1 |
|  | (2.3±0.1) | (4.9±0.2) | (8.5±0.3) | (8.7±0.2) | (8.4±0.2) | (6.2±0.1) | (6.5±1.0)^a^ |
| **eCO_2_** | 6.7±0.6 | 28.7±3.1 | 72.2±3.4 | 84.3±5.0 | 71.9±2.6 | 39.8±2.1 | 50.6±12.3 |
|  | (2.7±0.1) | (5.3±0.2) | (8.5±0.2) | (9.1±0.2) | (8.5±0.1) | (6.3±0.1) | (6.7±1.0)^a^ |
| **eO_3_** | 3.4±0.4 | 16.6±1.1 | 46.0±3.5 | 42.4±4.2 | 31.4±2.0 | 11.3±1.5 | 25.1±7.0 |
|  | (2.0±0.1) | (4.1±0.1) | (6.8±0.2) | (6.5±0.3) | (5.6±0.1) | (3.4±0.2) | (4.7±0.7)^c^ |
| **AM (Ambient)** | 4.9±0.5 | 22.1±1.3 | 56.4±2.3 | 57.6±2.9 | 48.1±3.7 | 26.8±1.4 | 35.9±8.7 |
|  | (2.4±0.1) | (4.7±0.1) | (7.5±0.1) | (7.6±0.1) | (6.9±0.2) | (5.2±0.1) | (5.7±0.8)^b^ |
| **Mean±SE** | 4.8±0.6 | 22.9±2.5 | 61.7±6.4 | 65.0±9.3 | 55.4±9.7 | 28.9±6.5 |  |
|  | (2.3±0.1)^e^ | (4.8±0.2)^d^ | (7.8±0.4)^a^ | (8.0±0.6)^a^ | (7.3±0.6)^b^ | (5.3±0.6)^c^ |  |

Treatments, F = (113.19), LSD = (0.23), P<0.001

Weeks, F = (450.7), LSD = (0.29), P<0.001

Interactions, F = (5.6), LSD = (0.59), P<0.001

Planthopper count with different subscript differ significantly

*Average of ten replication

Numbers in parenthesis are SQRT (X+1) valued

**Table 6. *N. lugens* nymphal population/hill in FATE during 2020**

|  | **Nymph population*** | | | | | | |
| --- | --- | --- | --- | --- | --- | --- | --- |
|  | **Weeks after adult release (WAR)** | | | | | | |
| **Treatments** | **1** | **2** | **3** | **4** | **5** | **6** | **Mean±SE** |
| **eT+eCO_2_** | 2.6±0.5 | 17.2±1.8 | 54.6±4.2 | 55.6±3.5 | 51.8±3.2 | 21.7±1.5 | 33.9±7.9 |
|  | (1.8±0.14) | (4.2±0.2) | (7.4±0.2) | (7.4±0.2) | (7.2±0.2) | (4.7±0.1) | (5.4±0.8)^a^ |
| **eCO_2_** | 4.6±0.6 | 21.2±2.3 | 50.2±3.0 | 61.3±4.1 | 50.3±2.5 | 24.9±1.8 | 35.4±7.5 |
|  | (2.3±0.1) | (4.6±0.2) | (7.1±0.2) | (7.8±0.2) | (7.1±0.2 | (5.0±0.2) | (5.6±0.7)^a^ |
| **eO_3_** | 2.6±0.4 | 11.4±1.3 | 31.8±2.4 | 27.8±2.9 | 18.1±2.4 | 5.9±1.6 | 16.2±4.0 |
|  | (1.8±0.1) | (3.4±0.18) | (5.6±0.2) | (5.3±0.2) | (4.2±0.27) | (2.4±0.3) | (3.8±0.5)^c^ |
| **AM (Ambient)** | 2.7±0.4 | 15.4±0.9 | 40.8±2.6 | 44.4±2.8 | 35.6±2.7 | 14.4±0.9 | 25.5±5.8 |
|  | (1.8±0.1) | (4.0±0.1) | (6.4±0.2) | (6.7±0.2) | (6.0±0.2) | (3.9±0.1) | (4.8±0.7)^b^ |
| **Mean±SE** | 3.1±0.3 | 16.3±1.5 | 44.3±3.9 | 47.2±5.7 | 38.9±6.0 | 16.7±3.2 |  |
|  | (1.9±0.1)^d^ | (4.0±0.2)^c^ | (6.6±0.3)^a^ | (6.8±0.4)^a^ | (6.1±0.5)^b^ | (4.0±0.4)^c^ |  |

Treatments, F = (98.5), LSD = (0.23), P<0.001

Weeks, F = (350.0), LSD = (0.28), P<0.001

Interactions, F = (5.9), LSD = (0.57), P<0.001

Planthopper count with different subscript differ significantly

*Average of ten replication

Numbers in parenthesis are SQRT (X+1) valued

**Table 7. *N. lugens* female population/hill in FATE during 2020**

|  | **Female population*** | | | | | | |
| --- | --- | --- | --- | --- | --- | --- | --- |
|  | **Weeks after adult release(WAR)** | | | | | | |
| **Treatments** | **1** | **2** | **3** | **4** | **5** | **6** | **Mean±SE** |
| **eT+eCO_2_** | 0.9±0.2 | 2.7±0.5 | 12.4±1.5 | 13.8±1.2 | 10.9±1.3 | 4.7±0.7 | 7.5±2.2 |
|  | (1.3±0.08) | (1.8±0.1) | (3.6±0.2) | (3.8±0.1) | (3.4±0.19) | (2.3±0.1) | (2.7±0.4)^b^ |
| **eCO_2_** | 1.1±0.27 | 2.7±0.7 | 11.9±1.59 | 14.4±2.0 | 11.7±1.0 | 9.8±0.9 | 8.6±2.2 |
|  | (1.4±0.09) | (1.8±0.18) | (3.5±0.2) | (3.8±0.2) | (3.5±0.1) | (3.2±0.1) | (2.9±0.4)^a^ |
| **eO_3_** | 0±0 | 3.2±0.49 | 10.7±1.2 | 8.1±1.0 | 8.2±1.0 | 3.5±0.5 | 5.6±1.6 |
|  | (1±0) | (2.0±0.1) | (3.3±0.19) | (2.9±0.1) | (2.9±0.1) | (2.1±0.1) | (2.4±0.3)^c^ |
| **AM (Ambient)** | 1.2±0.5 | 2.8±0.6 | 11.2±1.0 | 11.2±1.1 | 7.4±0.7 | 5.5±0.7 | 6.5±1.7 |
|  | (1.4±0.1) | (1.8±0.1) | (3.4±0.1) | (3.4±0.1) | (2.8±0.1) | (2.5±0.1) | (2.6±0.3)^b^ |
| **Mean±SE** | 0.8±0.2 | 2.8±0.1 | 11.5±0.3 | 11.8±1.4 | 9.5±1.0 | 5.8±1.3 |  |
|  | (1.2±0.1)^e^ | (1.9±0.03)^d^ | (3.4±0.05)^ab^ | (3.5±0.2)^a^ | (3.2±0.1)^b^ | (2.5±0.2)^c^ |  |

Treatments, F= (10.745), LSD= (0.17), P<0.001

Weeks, F= (135.076), LSD= (0.21), P<0.001

Interactions, F= (2.528), LSD= (0.43), P<0.001

Planthopper count with different subscript differ significantly

*Average of ten replication

Numbers in parenthesis are SQRT (X+1) valued

**Table 8. *N. lugens* male population/hill in FATE during 2020**

|  | **Male population*** | | | | | | |
| --- | --- | --- | --- | --- | --- | --- | --- |
|  | **Weeks after adult release (WAR)** | | | | | | |
| **Treatments** | **1** | **2** | **3** | **4** | **5** | **6** | **Mean±SE** |
| **eT+eCO_2_** | 1.0±0.2 | 4.4±0.6 | 5.4±0.8 | 6.5±0.7 | 7.8±1.0 | 11.5±1.1 | 6.1±1.2 |
|  | (1.3±0.09) | (2.2±0.1) | (2.4±0.1) | (2.7±0.1) | (2.9±0.1) | (3.5±0.1) | (2.5±0.2)^a^ |
| **eCO_2_** | 1.0±0.2 | 4.8±0.9 | 10.1±0.9 | 8.6±1.7 | 9.9±1.1 | 5.1±0.5 | 6.5±1.2 |
|  | (1.3±0.07) | (2.3±0.1) | (3.3±0.1) | (2.9±0.2) | (3.2±0.1) | (2.4±0.1) | (2.6±0.2)^a^ |
| **eO_3_** | 0.8±0.2 | 2.0±0.4 | 3.5±0.7 | 6.5±0.9 | 5.1±0.9 | 1.9±0.4 | 3.3±0.7 |
|  | (1.3±0.07) | (1.6±0.1) | (2.0±0.2) | (2.6±0.1) | (2.4±0.1) | (1.6±0.1) | (1.9±0.1)^c^ |
| **AM (Ambient)** | 1.0±0.2 | 3.9±0.4 | 4.4±1.0 | 2.0±0.5 | 5.1±0.9 | 6.9±1.0 | 3.8±0.7 |
|  | (1.3±0.07) | (2.1±0.1) | (2.2±0.2) | (1.6±0.1) | (2.4±0.1) | (2.7±0.1) | (2.1±0.1)^b^ |
| **Mean±SE** | 0.9±0.03 | 3.7±0.4 | 5.8±1.1 | 5.9±1.0 | 6.9±0.9 | 6.3±1.5 |  |
|  | (1.3±0.01)^c^ | (2.1±0.1)^b^ | (2.5±0.2)^a^ | (2.5±0.2)^a^ | (2.7±0.1)^a^ | (2.5±0.2)^a^ |  |

Treatments, F= (23.99), LSD= (0.18), P<0.001

Weeks, F= (38.2), LSZ= (0.22), P<0.001

Interactions, F= (6.6), LSD= (0.45), P<0.001

Planthopper count with different subscript differ significantly

*Average of ten replication

Numbers in parenthesis are SQRT (X+1) valued

**Photo of FATE experimental set up having rice plants kept inside FATE rings**


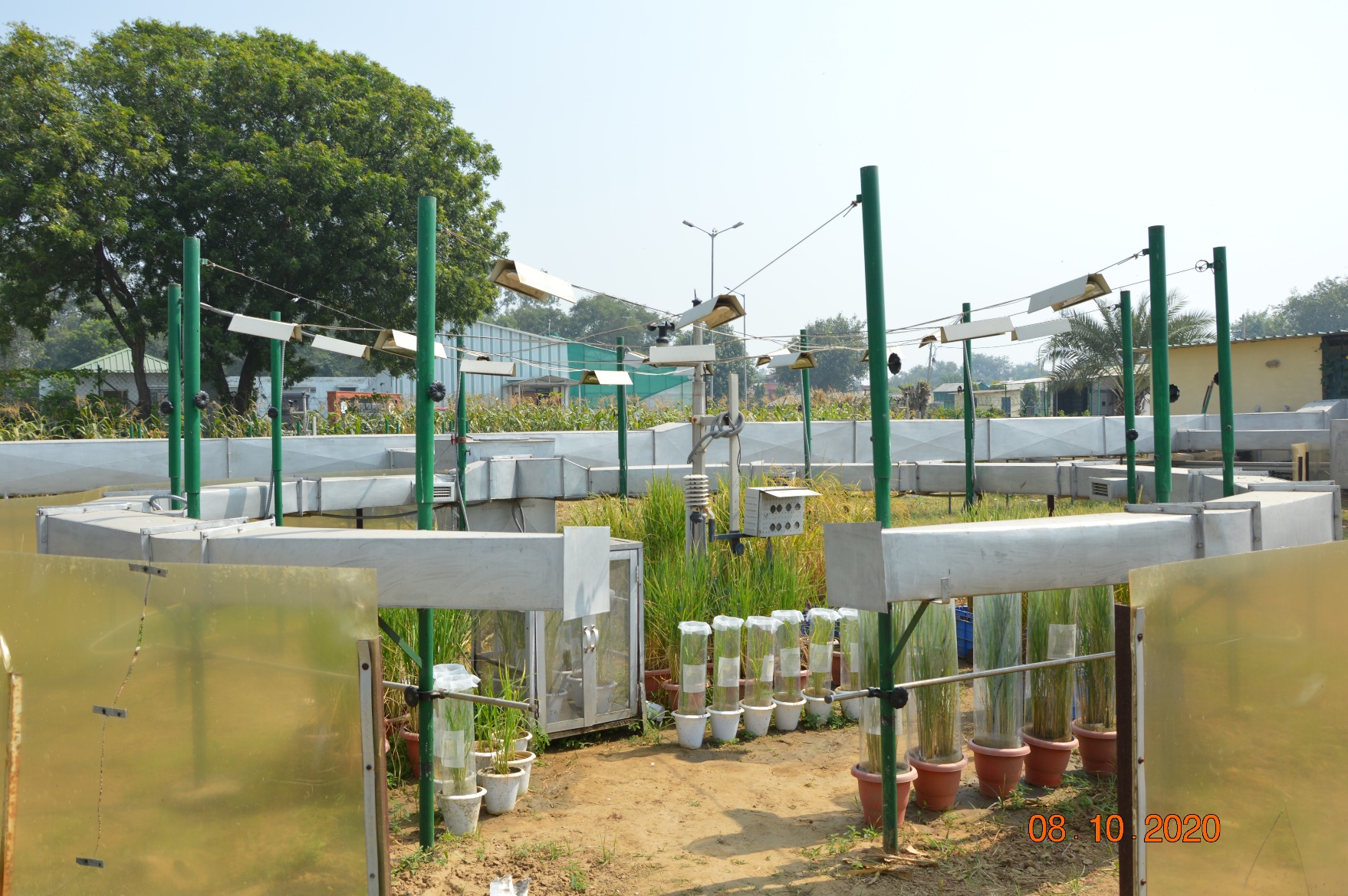

Supplement: Supplementary file 1 [file DataSheet1.docx]
